# Complex and Multifaceted Therapy-Related Myeloid Neoplasm Following Laryngeal Cancer Treated with Cisplatin and Radiotherapy

**DOI:** 10.4084/MJHID.2013.030

**Published:** 2013-04-15

**Authors:** Pasquale Niscola, Gianfranco Catalano, Andrea Tendas, Marco Giovannini, Laura Scaramucci, Benedetta Neri, Luciana Morino, Daniela Piccioni, Stefano Fratoni, Alessio Perrotti, Paolo de Fabritiis

**Affiliations:** 1 Hematology Unit, S. Eugenio Hospital, Rome, Italy.; 2 Pathology Department, S. Eugenio Hospital, Rome, Italy.

## Introduction

Therapy-related myeloid neoplasm, usually in the forms of myelodysplastic syndromes/acute myeloid leukemias (t-MDS/AML), are well-known secondary malignancies occurring in cancer patients who received chemo-radiotherapy regimens, especially those including alkylating agents and topoisomerase II inhibitors.^1^ The leukemogenic potential of other compounds, such as platinum drugs, is less clearly established,^2^ although a slightly increased risk of secondary AML has been reported after therapy for ovarian^3^ and testicular^4^ cancers. We describe the quite rare occurrence of a myelodysplastic/myeloproliferative neoplasm (MDS/MPN) with multiple accumulating genetic anomalies not usually found together as JAK2 V617F, IDH2 R172K and 7q- deletion, after exposure to radiotherapy and platinum based chemotherapeutic agents in a patient treated for laryngeal cancer.

## Case presentation

**Figure 1:**
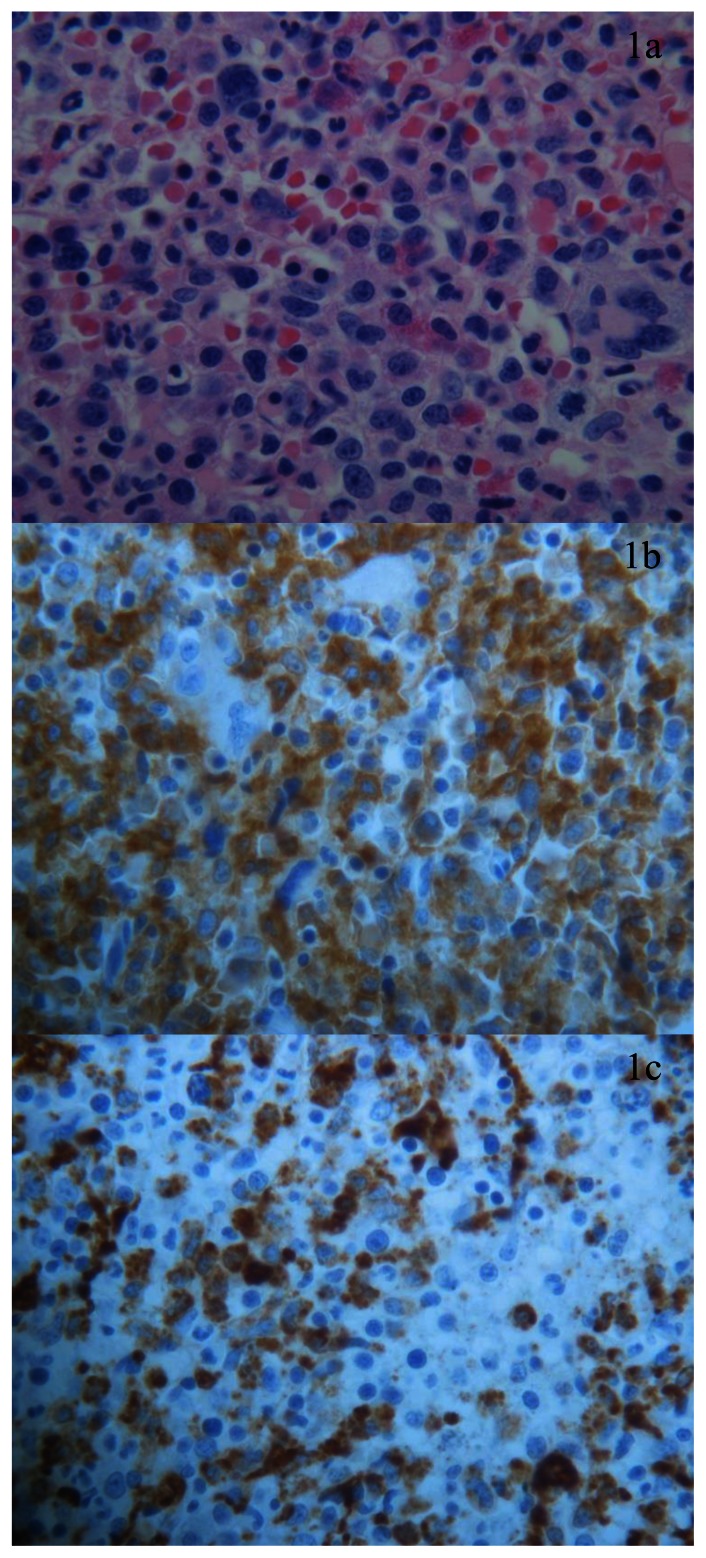
BM biopsy, high magnification (x 40) shows prominent myeloid proliferation (a); elements with myelomonocytic morphology expressing CD33 (b) and CD68RPGM1 (c) are prevalent. Dysplastic granulocytopoiesis and megakaryocytopoiesis are evident. Cells with undifferentiated immature morphology or blast equivalent as monoblast or promonocytes are very rare.

**Figure 2:**
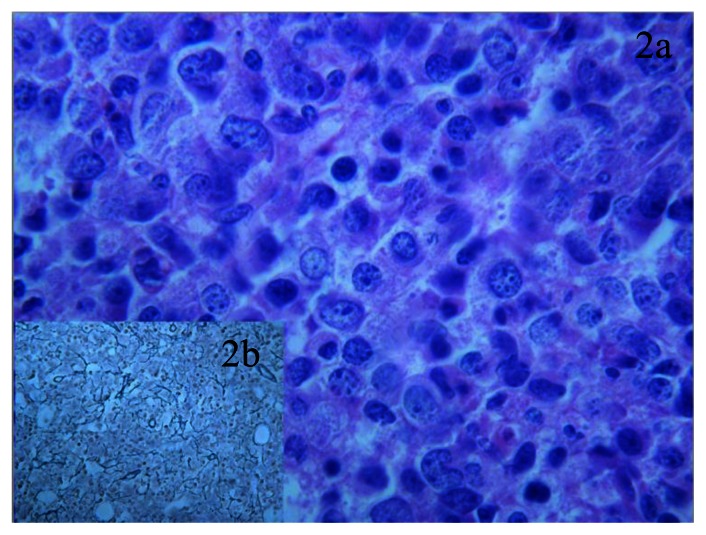
BM with large amount of monocytes and myeloid precursor such as promonocytes and cells with immature morphology featuring atypical nuclear anomalies, finely dispersed chromatin, small evident nucleoli and scant cytoplasm. Those cells are CD34 negative but express CD68KP1 and CD68RPGM1 (data not shown). The percentage of immature precursor through the entire BM biopsy has been estimated at about 15 – 20% of all hematopoietic cells. These findings were consistent with the diagnosis of chronic myelomonocytic leukemia- type2 (a). Insert b is a Gomori stain which shows a delicate bone marrow fibrosis, grade-1.

**Figure 3:**
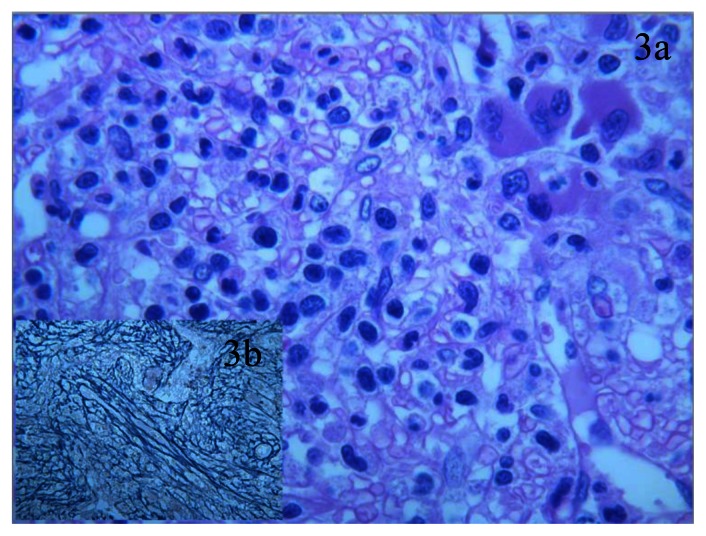
BM after azacitidine therapy reveals myelodysplastic features: dismegakariocytopoiesis and disgranulocytopoiesis are evident; rare erytroid islands with dyserythropoietic morphology are also detected (a). Cells with immature-blastic morphology are rare (less than 5%). Insert b: Gomori stain shows a diffuse and strong (grade-2) BM fibrosis.

A 60 years old man came to our attention on May 2011 because of isolated thrombocytopenia. He had been submitted to total laryngectomy for a laryngeal cancer, (May 2010) followed by local radiation (total dose: 6420 cGy) and chemotherapy. Cisplatin was given as a single agent with an adapted schedule (cumulative dose: 428 mg), due to a prolonged pancytopenia following administration of the first dose and an overall poor hematological tolerance. One year after chemotherapy the patient was referred to our outpatient clinic, due to mild asymptomatic thrombocytopenia (platelet count: 97.000/uL). Palpable splenomegaly, estimated by ultrasonography as of 20 cm longitudinal diameter, was present. Peripheral blood (PB) smears showed a pronounced anisocytosis and poikilocytosis of red blood cells (RBC) and platelets (PLTS) and the absence of morphological evidence of circulating blasts. Trephine biopsy revealed a hypercellular bone marrow (BM) indicative of refractory cytopenia with multilineage dysplasia (RCMD, figure 1a-c), mild and multifocal reticulin fibrosis (grade 1 according to European Consensus System)^5^ and about 3% of immature-blastic cells. Standard cytogenetic and FISH analysis on BM and PB showed no abnormalities (normal karyotype). BCR/ABL p210 and p190 molecular transcripts were not detectable, whereas a mutation of JAK2 V617F was present at quantitative PCR analysis. The diagnosis was of myelodysplastic/myeloproliferative neoplasm (MDS/MPN).^6^ Considering the temporal relation, we suspected a link with the previous cytotoxic treatments for laryngeal cancer. Ten months after the initial diagnosis of MDS/MPN, the patient presented a rapidly progressive spleen enlargement leading within few weeks to massive and painful splenomegaly. He received hydroxycarbamide without benefit. While his blood counts did progressively deteriorate, an increasing monocytosis was recorded. A hematological diagnostic revision was performed with histopathological diagnosis of CMML with 15% of blasts in the BM (Figure 2a,b). MD Anderson Prognostic Scoring System (MDAPS)^7^ was 4 (high risk). The patient received palliative local radiotherapy with significant benefit, due to the extremely high symptoms burden of massive splenomegaly (27 × 20 cm on CT scan evaluation). Immediately after he was scheduled to receive six cycles of azacitidine (75 mg/m^2^, schedule 5+2 days excluding weekends, each cycle every 4 weeks), in the light of favorable reported experiences^8^,^9^ and according to the approved indications. The patient was properly informed and gave his consent. After the fourth azacitidine course, PB counts significantly improved, and the monocytosis disappeared; BM trephine biopsy showed the near full disappearance of blast cells but progression of the fibrosis (Figure 3a,b). The spleen was once again massively enlarged; JAK2 V617F mutation was still present. The treatments were well tolerated and no major clinical complications were observed; in particular, only mild transfusion requirement was recorded. Meanwhile, a HLA-identical sibling donor was found in order to plan allogenic stem cells transplantation (HSCT). Unfortunately, soon after the completion of the sixth course of azacitidine, an overt evolution into AML (myelomonoblastic subtype) occurred. The patient presented marked leukocytosis (WBC = 120.000/ul; 70% myelomonoblastic cells positive for HLA-DR, CD4, CD13, CD15, CD33, CD64, CD45, CD34, CD56 and CD117). The kariotype by standard cytogenetic was: 46, XY, del (7)(q31)[7]/46,XY[13]; FISH analysis confirmed the deletion of the long arm of chromosome 7 in 80% of blastic cells. Molecular studies for the most frequent AML-related alterations (CBFb/MYH11, DEK/CAN, NPM1, FLT3, RUNX1/ETO) showed no abnormalities. Apart from the persistence of JAK2 V617F mutation, the analysis of most common alterations found in t-MDS/AML showed only an IDH2 R172K mutation.^10^ After the fourth azacitidine course, PB counts significantly improved, and the monocytosis disappeared; BM trephine biopsy showed the near full disappearance of blast cells but progression of the fibrosis (Figure 3a,b). The spleen was once again massively enlarged; JAK2 V617F mutation was still present. The treatments were well tolerated, and no major clinical complications were observed; in particular, only mild transfusion requirement was recorded. Meanwhile, a HLA-identical sibling donor was found in order to plan allogenic stem cells transplantation (HSCT). Unfortunately, soon after the completion of the sixth course of azacitidine, an overt evolution into AML (myelomonoblastic subtype) occurred. The patient presented marked leukocytosis (WBC = 120.000/ul; 70% myelomonoblastic cells positive for HLA-DR,CD4,CD13,CD15,CD33,CD64, CD45,CD34,CD56 and CD117). The kariotype by standard cytogenetic was: 46, XY, del (7)(q31)[7]/46,XY[13]; FISH analysis confirmed the deletion of the long arm of chromosome 7 in 80% of blastic cells. Molecular studies for the most frequent AML-related alterations (CBFb/MYH11, DEK/CAN, NPM1, FLT3, RUNX1/ETO) showed no abnormalities. Apart from the persistence of JAK2 V617F mutation, the analysis of most common alterations found in t-MDS/AML showed only an IDH2 R172K mutation. The patient subsequently underwent to a chemotherapeutic treatment AML-like (FLA regimen) in order to submit him to a subsequent allogeneic bone marrow transplantation. However, the disease was resistant, and the patient died for pulmonary complications.

## Conclusion

Cisplatin is a DNA damaging drug, which exerts his cytotoxic properties by binding nuclear DNA, and then the most sensitive region of the genome such as telomeres interfering with normal transcription and replication mechanisms.^11^ DNA repair capacity, apart from being a major factor in conferring cisplatin resistance, may as well save partly damaged hemopoietic stem cells, giving rise to neoplastic clones. In addition, non-DNA targets, such as proteins, may contribute to cytotoxic effects induced by this agent.^12^ Although well described in the literature, therapy-related myeloid neoplasm after platinum-based chemotherapy and low dose local radiotherapy for solid tumors represents a rare occurrence.^2^ Within one year after he received cisplatin, our patient was diagnosed with an unusual MDS/MPN disorder in prefibrotic phase, characterized by a hypercellular BM and minimal fibrosis; the hematological features of the disease in a short time changed in an accelerated and “subacute” fashion until full development of CMML. As secondary and therapy-related neoplasm, the latter has been only occasionally reported in the settings of solid tumors^13^,^14^ and hematological malignancies.^15^,^16^ A true link between the antineoplastic treatments for laryngeal cancer and the close onset of the atypical MDS/MPN disorder is difficult to assess; however, we consider this relationship at least conceivable due to the time course of events. The second course of radiotherapy on the spleen could have had a role in multiplying the damage initiated by cisplatin and the first course of radiotherapy. One can speculate that the second course of radiotherapy on the spleen stopped the repair of DNA sequence, easing the occurrence of the secondary leukemic event.

The sequence of events could be: Development of secondary JAK2 V617F myelomonocitic neoplasm, transformation in a more aggressive form as CMML and finally AML with IDH2 mutation and 7q-deletion.

Leukemic transformation of chronic MPNs is well known and relevant in the clinic, but very little is known about its molecular mechanisms of progression. We know that a substantial proportion of patients with JAK2V617F-mutant MPNs turns into a JAK2V617F-negative secondary AML (sAML). In our case the proliferative stimulus provided by JAK2 constitutively active kinase never defaulted, not even after hypomethylating therapy. Regarding IDH1/2 mutations, those are commonly identified in secondary gliomas that arise from a preceding lower-grade astrocytoma, suggesting a role in the progression to high-grade neoplasms.^17^ IDH2 is the mitochondrial form of the three isocitrate dehydrogenase genes (IDH1-2-3).^18^–^20^ That anomaly gains to the enzyme the function of 2-hydoxiglutarate production with methabolic implications yet not well elucidated. It seems to be relevant for cancer cell proliferation, may be through altering cellular respiration, and has been associated with unfavorable prognosis in AML.^21^ In conclusion, we have reported on a patient with a likely therapy-related MDS/MPN, which initially appeared in the form of a prefibrotic atypical myeloid neoplasm with JAK2 V617F, rapidly transformed into a CMML and then in AML with multiple anomalies, all associated with an adverse prognosis, after a transient, brief response to epigenetic therapy.
